# Clinical improvement of Long-COVID is associated with reduction in autoantibodies, lipids, and inflammation following therapeutic apheresis

**DOI:** 10.1038/s41380-023-02084-1

**Published:** 2023-05-02

**Authors:** Martin Achleitner, Charlotte Steenblock, Juliane Dänhardt, Natalia Jarzebska, Romina Kardashi, Waldemar Kanczkowski, Richard Straube, Roman N. Rodionov, Nitzan Bornstein, Sergey Tselmin, Frank Kaiser, Ronald Bucher, Mahmoud Barbir, Ma-Li Wong, Karin Voit-Bak, Julio Licinio, Stefan R. Bornstein

**Affiliations:** 1 Department of Internal Medicine III, University Hospital Carl Gustav Carus, Technische Universität Dresden, Dresden, Germany; 2 Zentrum für Apherese- und Hämofiltration am INUS Tageklinikum, Cham, Germany; 3 Alpstein Clinic, Gais, Switzerland; 4 Biologicum Baden-Baden INUSpherese Zentrum, Baden-Baden, Germany; 5 Department of Cardiology, Harefield Hospital, Harefield, United Kingdom; 6 Department of Psychiatry and Behavioral Sciences, College of Medicine, State University of New York (SUNY) Upstate Medical University, Syracuse, NY USA; 7 Department of Neuroscience & Physiology, College of Medicine, SUNY Upstate Medical University, Syracuse, NY USA; 8 School of Cardiovascular and Metabolic Medicine and Sciences, Faculty of Life Sciences & Medicine, King’s College London, London, UK; 9 Department of Endocrinology, Diabetology and Clinical Nutrition, University Hospital Zurich (USZ), and University of Zurich (UZH), Zurich, Switzerland

**Keywords:** Diagnostic markers, Biological techniques

## Abstract

In the aftermath of the COVID-19 pandemic, we are witnessing an unprecedented wave of post-infectious complications. Most prominently, millions of patients with Long-Covid complain about chronic fatigue and severe post-exertional malaise. Therapeutic apheresis has been suggested as an efficient treatment option for alleviating and mitigating symptoms in this desperate group of patients. However, little is known about the mechanisms and biomarkers correlating with treatment outcomes. Here, we have analyzed in different cohorts of Long-Covid patients specific biomarkers before and after therapeutic apheresis. In patients that reported a significant improvement following two cycles of therapeutic apheresis, there was a significant reduction in neurotransmitter autoantibodies, lipids, and inflammatory markers. Furthermore, we observed a 70% reduction in fibrinogen, and following apheresis, erythrocyte rouleaux formation and fibrin fibers largely disappeared as demonstrated by dark field microscopy. This is the first study demonstrating a pattern of specific biomarkers with clinical symptoms in this patient group. It may therefore form the basis for a more objective monitoring and a clinical score for the treatment of Long-Covid and other postinfectious syndromes.

## Introduction

Following viral infections, post-infectious complications may occur and trigger debilitating disease [1, 2]. This has occurred with the COVID-19 pandemic in an unprecedented and unparalleled way leaving millions of people in despair. Patient self-help organizations have mushroomed around the globe demanding from political stakeholders and health authorities immediate action. Post-Covid clinics have been established in some centers trying to provide some comfort but currently have not been able to offer much for an increasingly frustrated group of patients and their family members left alone with their misery.

Therapeutic apheresis has been reported to be effective in many patients affected [3–6] but due to the lack of validated controlled trials and high treatment costs, health insurance companies have rarely provided reimbursement. Because of the lack of controls and single reports in the media of patients showing no improvement after therapeutic apheresis, critical papers questioning any benefit of such an invasive procedure have been published [7, 8]. Randomized controlled trials using an invasive procedure requiring sham treatments are ethically challenging and time-consuming due to regulatory issues. This underlines the need for approving practice-oriented medical approaches instead of insisting on evidence-based medicine in such times of immediate need.

Current mechanisms suggested to explain the sequelae of chronic fatigue and post-exertional malaise in patients with Long-Covid include reduced tissue perfusion [9], viral infiltration of tissues, inflammation in the brain and peripheral organs [10], the persistence of SARS-CoV-2 spike proteins [11], and reactivation of other infectious agents including Epstein-Barr Virus, CMV and other infectious components [12–16]. Furthermore, recent data have demonstrated that there is a major rise in autoimmune diseases following COVID-19 [17–19] due to the generation of known and hitherto unknown autoantibodies [20]. For example, neurotransmitter autoantibodies and other autoantibodies have been suggested to play a role in COVID-19 severity [21] and Long-Covid [20, 22]. Finally, alteration of blood cellular components [23, 24] and rarefication of vessels [25] have been suggested to play a role in the development of Long-Covid following infection with SARS-CoV-2. Recent evidence has clearly shown that elevated lipids constitute a major risk factor [26–28]. Similarly, Long-Covid triggers a significant increase in lipids causing long-term risk for cardiovascular disease [29].

Based on these findings, there is a clear rationale for extracorporeal apheresis in patients with Long-Covid. Extracorporeal apheresis and immune adsorption are well-established methods for reducing all major lipids including ceramides, LP(a), and inflammatory lipids that cannot be lowered by statins or other medications [30–33].

Secondly, apheresis improves blood flow and has been used for autoimmune and inflammatory diseases of the nervous system and brain [34]. Therefore, this study aimed to elucidate the role of potential biomarkers in relation to treatment outcomes in Long-Covid patients undergoing therapeutic apheresis. This should form the basis for allowing a risk-benefit strategy of who should receive treatment and a set of profiles of markers monitoring treatment success.

## Material and methods

### Patients and assessment of Long-Covid symptoms

From the Long-Covid outpatient department at the University Clinic in Dresden in Germany 123 patients, which reported having fatigue symptoms for at least twelve weeks, were included. A standardized questionnaire FACIT-F (https://www.facit.org/measures/FACIT-F) approved for the assessment of fatigue in autoimmune diseases [35] was used to measure the degree of fatigue in these patients. Moreover, a visual analog scale was used to determine tiredness.

In Cham and Baden-Baden in Germany and at the Alpstein Clinic in Gais in Switzerland, a cohort of 27 patients with Long-Covid was treated with therapeutic apheresis and chronic fatigue syndrome and post-exertional malaise were evaluated by standard questionnaires for Long-Covid before and after apheresis. Informed consent was obtained from all study subjects.

### Therapeutic apheresis (INUSpheresis)

A cohort of 27 patients with Long-Covid was treated with a filtration-based (TKM58) therapeutic apheresis approach, specifically INUSpheresis, which is known to remove autoantibodies, inflammatory cytokines, oxidated LDLs, environmental toxins, and large molecules, contributing to plasma viscosity [5]. The patients were treated twice at an interval of 3 weeks. Each treatment lasted an average of 114 min and the patients received 8000E Heparin per treatment.

### Biomarker measurements

Blood was collected from the Long-Covid patients pre- and post-apheresis. Inflammatory factors (sCRP, IL-1beta, and IL-6), autoantibodies (against ß-adrenergic receptors and acetylcholine receptors), lipids (chol, TG, LDL, HDL, and Lp(a)), thrombotic factors (fibrinogen and homocysteine), and H_2_O_2_ were measured. Antibodies against α_1_- and β_1_-adrenergic receptors and the muscarinic acetylcholine receptors 3 and 4 were measured with commercially available immunoassays (CellTrend GmbH, Luckenwalde, Germany) according to the instructions of the manufacturer. The remaining factors were measured with standardized commercial kits.

### Microscopy

Erythrocytes were evaluated microscopically pre- and post-apheresis using a NIKON ECLIPSE E 200 microscope.

## Results

### Neurotransmitter autoantibodies in Long-Covid patients with fatigue

One aim of the study was to investigate whether increased fatigue symptomatic after SARS-CoV-2 infection is associated with elevated antibody titers against certain neurotransmitter receptors. Therefore, we measured the levels of 4 different auto-antibodies (β1 and β2 adrenergic receptors (AdR), M3 and M4 acetylcholine receptors (AChR)) in 123 patients that reported fatigue symptoms for at least 12 weeks after COVID-19. It turned out that there was a very high antibody titer variation between patients and no direct correlation between symptom severity and the levels of antibodies. The mean level of autoantibodies against the ß1-AdR was not increased in comparison to the reference level, whereas the other 3 autoantibodies (ß2-Adr, M3 AChR, and M4 AChR) were increased compared to reference levels (Table 1). These data suggest that an increase in autoantibodies against one neurotransmitter does not lead to Long-Covid fatigue symptoms, but that an increase in at least 2-3 different autoantibodies may be required for symptom development.

**Table 1:** Neurotransmitter autoantibody titers.

Antibody	ß1-adr-R-Ab (U/ml)	ß2-adr-R-Ab (U/ml)	M3 R-Ab (U/ml)	M4 R-Ab (U/ml)
Long-Covid patients	12.18 ± 10.97	20.75 ± 16.13	18.18 ± 15.31	11.68 ± 9.01
Reference value	15	14	10	10.7

Antibody titers were measured in the blood of Long-Covid patients with reported fatigue (*n* = 123).

### Characteristics of apheresis patients

Previously, we have described that after 3 treatments with INUSpheresis, 74% of 1111 patients with ME/CFS, including a subgroup (13%) of Long-Covid patients, reported having no symptoms or feeling significantly better, 11% reported moderate improvement, and 15% didn’t feel better 6 months after therapeutic apheresis based on currently used assessment parameters for Long-Covid [5]. To investigate whether this improvement is caused by a reduction of biomarkers associated with Long-Covid, we performed INUSpheresis on 27 severely affected Long-Covid patients (14 men, 13 women) and measured several biomarkers in the blood before and after 2 therapeutic apheresis treatments. The mean age of the men was 49.7 years and of the women, it was 44.9 years. The men had a mean BMI of 26.02 kg/m^2^ and the women of 21.72 kg/m^2^. The main symptoms of Long-Covid in this patient group were immunopathies (65%), ME/CFS (52%), intestinal dysbiosis (28%), and polyneuropathies (21%).

### Biomarker screening in Long-Covid patients

To test whether therapeutic apheresis may lead to a reduction of biomarkers associated with Long-Covid, we tested the concentrations of these markers in the blood of Long-Covid patients pre- and post-extracorporeal therapeutic apheresis.

**Fig. 1:**
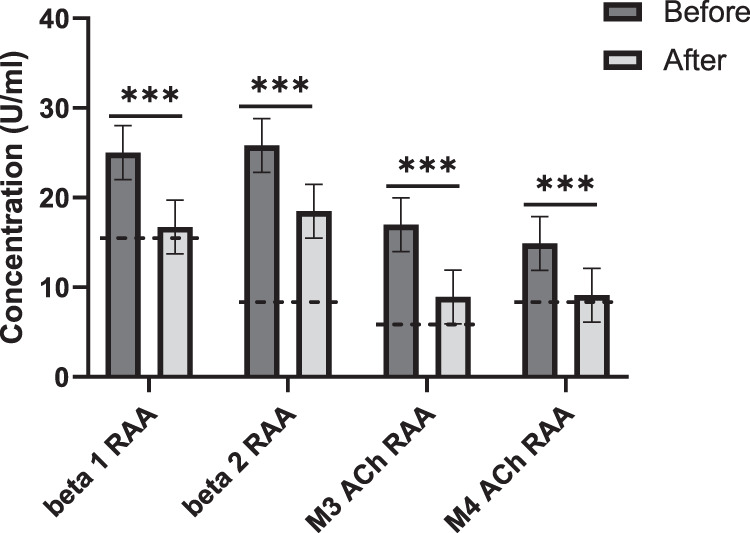
Neurotransmitter autoantibodies are reduced after apheresis. Antibodies against the β1 and β2 adrenergic receptors (AdR) and the M3 and M4 acetylcholine receptors (AChR) were measured in the blood of Long-Covid patients (*n* = 27) pre- and post-apheresis. Reference values are marked with a dotted line. RAA: receptor autoantibodies. ***P < 0.001 (paired two-sided *t*-test).

First, we measured the autoantibodies against β1- and β2-AdR and against M3- and M4-AChR. In contrast to the data in Table 1, also the antibodies against ß1-AdR were increased in these patients before apheresis. Also, the autoantibodies against ß2-AdR, and M3- and M4-AChR were increased compared to reference levels. Autoantibodies against ß1- and ß2-AdR were decreased by 33% and 28%, respectively, whereas autoantibodies against M3- and M4-AChR were reduced by 48% and 39%, respectively, after apheresis (Fig. 1).

**Fig. 2:**
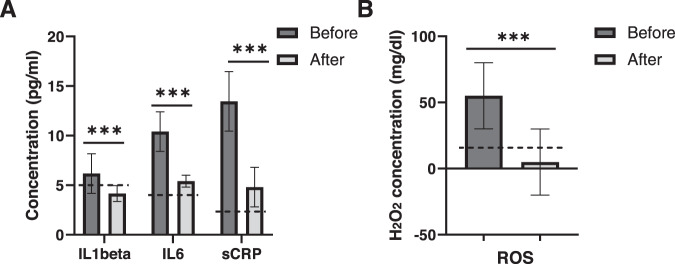
Inflammatory and oxidative factors are reduced after apheresis. IL-1beta, IL-6, sCRP (**A**), and H_2_O_2_ (**B**) were measured in the blood of Long-Covid patients (*n* = 27) pre- and post-apheresis. Reference values are marked with a dotted line. ***P < 0.001 (paired two-sided *t*-test).

Furthermore, sCRP and the pro-inflammatory cytokines IL-1 beta and IL-6 were all significantly reduced after apheresis (33%, 48%, and 64%) (Fig. 2A). As oxidative stress has been shown to be associated with Long-Covid, we measured the concentration of H_2_O_2_ in the blood and observed a more than 90% decrease after 2 rounds of therapeutic apheresis (Fig. 2B).

**Fig. 3:**
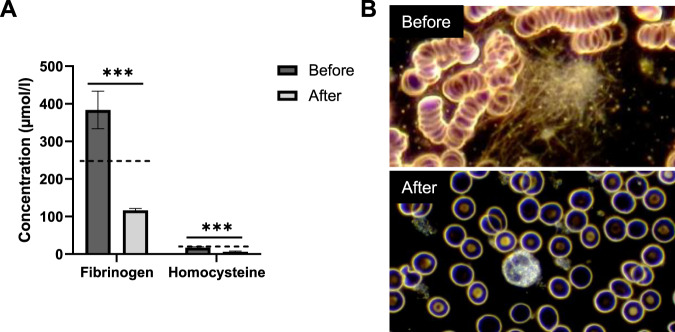
Apheresis reduces factors related to rheology. **A** Fibrinogen and homocysteine were measured in the blood of Long-Covid patients (*n* = 27) pre- and post-apheresis. Reference values are marked with a dotted line. ***P < 0.001 (paired two-sided *t*-test). **B** Erythrocytes were imaged before and after apheresis. Representative pictures are shown.

Factors related to rheology, such as fibrinogen and homocysteine, were significantly reduced by 70% and 64%, respectively (Fig. 3A). Both factors reached levels below the reference value. This was confirmed microscopically, as it could be observed that in some patients, the erythrocytes in Long-Covid aggregated in closely packed stacks of cells, forming three-dimensional structures, so-called rouleaux (Fig. 3B). Furthermore, the blood was characterized by fibrin fibers and sediments (Fig. 3B). After apheresis, the rouleaux formation had vanished and fibrin fibers were gone (Fig. 3B).

**Fig. 4:**
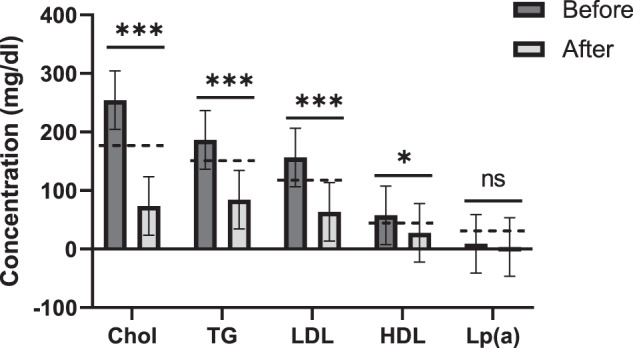
Apheresis reduces lipids levels. Lipids were measured in the blood of Long-Covid patients (*n* = 27) pre- and post-apheresis. Reference values are marked with a dotted line. ns *P* > 0.05; *P < 0.05; ***P < 0.001 (paired two-sided *t*-test).

When we measured lipids in the blood, we observed that cholesterol, triglycerides (TG), LDL, and HDL, were all significantly reduced in an expected manner (Fig. 4). All lipids reached values below the reference value.

## Discussion

Extracorporeal apheresis has the potential to improve symptoms of Long-Covid. Consistent with the clinical experience of several centers that have administered this treatment in a cohort of more than 1000 patients, 70% of patients with ME/CFS, including Long-Covid patients, reported a significant improvement in their symptoms [5]. In the current study, we analyzed those biomarkers that play a role in the pathogenesis of postinfectious syndromes, such as Long-Covid, in 27 patients who showed clinical improvement after extracorporeal therapeutic apheresis and found that the biomarkers were significantly reduced after the treatment.

A limitation of our study is that it was not conducted as a randomized controlled trial that included sham experiments of the invasive procedure without therapeutic filters. Optimally, such sham experiments should be included to exclude that the benefit of apheresis is mediated by biological factors, e.g. heparin, which promotes tissue perfusion. Undoubtedly, there is a need to perform larger randomized controlled trials. However, there are several problems with performing large-scale randomized controlled trials. First, patients with Long- or Post-Covid are a heterogeneous group of patients, and inclusion criteria for a controlled trial will be difficult. This becomes evident in our study of different cohorts diagnosed in Post-Covid clinics at our centers that used stringent currently used qualitative assessment criteria for Long-Covid symptoms. Thus, patients with classical symptoms of chronic fatigue including severe exhaustion, tiredness, post-exertional malaise, depression, headache, tinnitus, muscle pain, abdominal pain, and brain fog with typically reduced strengths in handgrips, still demonstrated a variety in the pattern of altered biomarkers. Some patients with substantially elevated neurotransmitter antibodies and only mild inflammation were showing severe symptoms. Similarly, patients with normal autoantibodies but significantly elevated lipids, inflammatory markers, and signs of oxidative stress also suffered from severe forms of chronic fatigue. Therefore, given the fact that chronic fatigue following COVID-19 seems to be a multi-faceted disease with diverse potential disease mechanisms, it will be unlikely that we will find one single biomarker correlating with the clinical symptoms of these patients. Most likely, we will have to develop biomarker scores as it is routinely done in other forms of autoimmune diseases, e.g. rheumatoid arthritis. This will provide useful monitoring of treatment success or failure. It will however be difficult to establish a clear-cut correlation and causality.

Finally, current clinical protocols in different centers, including the one presented here, have been using a great variety of procedures not only including therapeutic apheresis but also antioxidative substances, and anti-inflammatory treatment, which may or may not include glucocorticoids. These components all may have an impact on treatment success [3–6]. Therefore, a controlled trial generating clear evidence would be enormously complex to perform.

Before apheresis treatment, it became apparent that some Long-Covid patients had high levels of erythrocyte aggregation, which is common in diseases associated with hyperthermia and hypoxemia, such as stroke or COVID-19 [36]. Elevated blood lipids, as observed in Long-Covid [27, 29], may also increase the risk of cardiovascular diseases highlighting the necessity of lowering both lipids and thrombotic factors [37]. Extracorporeal therapeutic apheresis was initially established for the reduction of lipids in severe dyslipidemias. Later studies showed that this treatment has several additional beneficial effects as high molecular weight proteins are reduced, blood viscosity is improved, oxidative stress is reduced, and cytokines and autoantibodies are removed [30–33]. These results we confirmed in the current study, where all these biomarkers were significantly reduced in the blood of Long-Covid patients. The advantage of therapeutic extracorporeal apheresis in comparison to specific medication is its ability to remove a broad range of toxins, inflammatory proteins, lipids, and autoantibodies from human blood [32, 38, 39].

Increased inflammation and oxidative stress have been suggested as the main factors leading to fibrosis, thrombosis, autonomic nervous system dysfunction, and autoimmunity resulting in tissue damage and consequently Long-Covid [40, 41]. Therefore, the fact that we see both a decrease in the inflammatory factors CRP, IL-1beta, and IL-6 and in reactive oxygen species after apheresis might at least partly explain the reduction in symptoms reported by the patients.

As for ME/CFS, antibodies against G-protein coupled receptors (GPCRs), such as adrenergic and muscarinic receptors, were also detected in Long-Covid patients [22, 42]. In the current study, we observed a reduction in four such GPCR-autoantibodies post apheresis. The antibodies were not completely removed but this reduction might be enough to reduce symptoms as previous studies showed that in a subgroup of ME/CFS patients, B-cell depletion with rituximab was associated with symptom improvement [43, 44]. Furthermore, it is important not to remove all antibodies from the blood as an adequate immune response against SARS-CoV-2 and other pathogens should be maintained. Apheresis likely reduces further autoantibodies that have not yet been defined but are involved in the pathology of Long-Covid symptoms.

In conclusion, this study clearly shows that extracorporeal apheresis is a powerful technology to reduce biomarkers that have been implicated in the pathogenesis of post-infectious syndromes such as Long-Covid. This, however, describes only an association and not causality and a clear correlation with symptoms and improvement of patients. Therefore, an additional limitation of our and similar studies is the current lack of hard endpoints for clearly defining and measuring improvements in Long-Covid symptoms objectively. Nevertheless, the study provides some guidance for monitoring treatment success and for establishing a larger patient cohort with a score of several of these parameters that will correlate with the immediate and long-term outcomes of the procedure. We believe that in the acute situation of such a mass number of desperate individuals suffering from a multifaceted disorder such as Long-Covid entirely relying on the most stringent criteria of evidence-based medicine did not and will not fully meet the needs of these patients.

This therapeutic option has to be presented honestly and transparently to patients seeking help and improvement of their current situation. It needs to be acknowledged that this treatment will not be successful in every patient and that we do not fully understand the exact mechanisms of treatment success or failure at this point. There has to be room for practice-oriented medicine that allows providing this treatment to those patients in need while the performance of controlled randomized evidence-based trials is needed. Private centers engaged in this type of treatment have to be involved in the monitoring of their patients. In such a complex disorder with a combination of diverse pathological mechanisms, individualized trials with different treatment protocols will be necessary. Extracorporeal therapeutic apheresis has been used for many decades and is an extremely safe method but nevertheless an invasive procedure and patients have to be informed about rare complications. Modern artificial intelligence-based technologies involving machine learning will be ideally suited to design and define individual treatment protocols with specific markers for the different patient groups of post-infectious syndromes in the future.

To develop a future clinical score, multicenter studies with a larger number of patients, including those that have shown no improvement with therapeutic apheresis, will have to be performed to correlate individual biomarkers with the outcome of treatment success. Specifically, multivariate analysis will be necessary to establish a useful clinical score for monitoring initial and long-term treatment success. Certainly, a better exploration of disease mechanisms for Long-Covid and other postinfectious syndromes may improve or add to such a panel of clinical biomarkers.
